# Capacity Limit of Simultaneous Temporal Processing: How Many Concurrent ‘Clocks’ in Vision?

**DOI:** 10.1371/journal.pone.0091797

**Published:** 2014-03-14

**Authors:** Xiaorong Cheng, Qi Yang, Yaqian Han, Xianfeng Ding, Zhao Fan

**Affiliations:** 1 Key Laboratory of Adolescent Cyberpsychology and Behavior (CCNU), Ministry of Education, Wuhan, China; 2 School of Psychology, Central China Normal University, Wuhan, China; 3 Key Laboratory of Human Development and Mental Health of Hubei Province, Wuhan, China; University of Muenster, Germany

## Abstract

A fundamental ability for humans is to monitor and process multiple temporal events that occur at different spatial locations simultaneously. A great number of studies have demonstrated simultaneous temporal processing (STP) in human and animal participants, i.e., multiple ‘clocks’ rather than a single ‘clock’. However, to date, we still have no knowledge about the exact limitation of the STP in vision. Here we provide the first experimental measurement to this critical parameter in human vision by using two novel and complementary paradigms. The first paradigm combines merits of a temporal oddball-detection task and a capacity measurement widely used in the studies of visual working memory to quantify the capacity of STP (CSTP). The second paradigm uses a two-interval temporal comparison task with various encoded spatial locations involved in the standard temporal intervals to rule out an alternative, ‘object individuation’-based, account of CSTP, which is measured by the first paradigm. Our results of both paradigms indicate consistently that the capacity limit of simultaneous temporal processing in vision is around 3 to 4 spatial locations. Moreover, the binding of the ‘local clock’ and its specific location is undermined by bottom-up competition of spatial attention, indicating that the time-space binding is resource-consuming. Our finding that the capacity of STP is not constrained by the capacity of visual working memory (VWM) supports the idea that the representations of STP are likely stored and operated in units different from those of VWM. A second paradigm confirms further that the limited number of location-bound ‘local clocks’ are activated and maintained during a time window of several hundreds milliseconds.

## Introduction

The ability of simultaneous temporal processing [1]–[8] can be life-critical for human. For example, a lifeguard with responsibility for a swimming pool full of children has to adopt a strategy of singling out a potential hazard by estimating how long a child has submerged. Since the children spread around the pool, it is necessary to establish individual-based temporal estimation at each child’s location and monitor multiple locations simultaneously. Besides, the pool and its vicinity are full of irrelevant distractors, such as moving adults, person’s shouting, laughing and water splashes. The mechanisms of how the lifeguard ignores those distractors and accomplishes the simultaneous temporal task to spot out a potential hazard are not yet fully understood.

Visual adaptation studies [9]–[11] shed some light on the property of the simultaneous temporal processing in vision by demonstrating that the encoding of visual duration operates in a spatially localized way. For example, temporal estimation to a 10 Hz grating of 600 ms duration (tester) was significantly compressed by a 15 s adaptation of an oscillating 20 Hz grating presented on the same part of the retina as the tester. This finding implicated that the multiple, independent time estimators/‘clocks’ of the STP might operate in a retinotopic [9]–[11] or spatialtopic [12] way, i.e., a time-space binding in visual modality.

Consider the predominant theory of time perception, i.e., the pacemaker-accumulator model [13] for a moment. This model posits that the duration encoding is accomplished by a ‘clock’-like structure, consisting of a pacemaker and an accumulator. The elapsed time is psychologically represented by the arithmetic summation of pulses emitted from the pacemaker at a regular pace, which are stored and added in the accumulator. In the context of this model, the most parsimonious way to time multiple durations concurrently is to have independent pulse making and accumulating units for those ‘local clocks’ [5]. This idea has been proposed, tested and confirmed in several studies of simultaneous temporal processing [1]–[8] in both animals and humans (but see [14] for a single clock strategy that might apply in simple tasks requiring minimum cognitive resource). Indeed, parallel timing across the visual and auditory modalities had been observed in a study with human participants [1] by using a stop-reaction-time (stop-RT) paradigm. Similarly, a recent research [8], using a temporal reproduction task on human, demonstrated that two independent clocks are involved in the timing of two multi-second intervals that are presented simultaneously in the visual modality. However, it is not clear so far how many independent timers/‘clocks’ are operated concurrently in vision at most. The majority literatures of simultaneous temporal processing used temporal intervals at multi-second level and did not measure the capacity of STP. In this study, we are interested in the capacity magnitude of STP at a time scale of sub-second level since previous literature [15] had revealed that the mechanisms of visual estimations between sub-second and supra-second levels are different and the former involves more automatic processing.

Another research line [16] indicated the necessity of spatial attention in visual temporal processing. For example, by using a dual-task paradigm, Cicchini and Morrone [16] found a perceived temporal compression by up to 40% to sub-second temporal intervals when attention was divided spatially. This finding implied that the operation of each ‘local clock’ of STP might require some attentional resource. Thus it is legitimate to ask how many location-bound ‘clocks’ can be activated and maintained simultaneously in vision when full attention is available, i.e., the spatial capacity of simultaneous temporal processing in vision. To our knowledge, up to now, there has been no study to directly measure this important capacity in visual modality.

The aim of the present study was to: (a) measure directly the capacity of simultaneous temporal processing (CSTP) in sub-second level and to elucidate the properties of the location-bound multiple ‘clocks’, including (b) whether the CSTP is constrained by other critical cognitive limitations, such as capacity of an individual’s visual working memory (VWM), and (c) whether spatial attention plays a critical role in the binding between a ‘local clock’ and its spatial location. We addressed these questions by using two novel and complementary paradigms, i.e. a temporal oddball-detection task to measure CSTP directly and a two-interval temporal comparison task to test an alternative account of CSTP measured by the first task.

## Experiment 1a

### Materials and Methods

Twenty Nine paid participants (24 female, 5 male; mean age = 21 years) from Central China Normal University (CCNU) took part in Experiment 1a & 1b. Procedures for all experiments in this study were approved by the institutional review board of CCNU. All participants in this study were right-handed, and naïve to the aim of this research. All of them had normal or corrected-to-normal vision, and had no history of neurological disorders or color blindness. All experiments in this study had been approved by the Research Ethics Committee of CCNU, and participants gave their written informed consent to participate.

The displays of all the experiments were programmed at a spatial resolution of 1024*768 pixels and a refresh rate of 100 Hz. The stimuli were displayed on an IIYAMA HM903DT color monitor, driven by a NVIDIA GeForce FX 5200 Graphics Adapter. Participants’ responses were recorded via a keyboard connected to the PC with their head stabilized by a chin rest and a viewing distance of 57 cm. The displays of all the experiments in this study were programmed in MATLAB (MathWorks Inc.) and Psychophysics Toolbox [17], [18].

**Figure 1:**
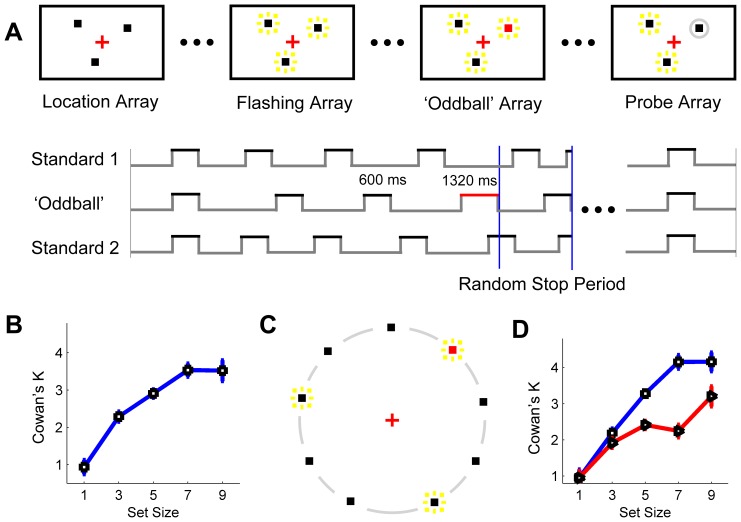
Experimental approach and results of Experiment 1a, 2 and 3. **A**, Temporal-oddball task (an exemplary trial of set size = 3; upper panel for stimuli setup, lower panel for timing traces). Red square denotes oddball and did not appear in the experimental display, so did for the yellow rectangles (denoting the repeated sequences of onsets and offsets of the squares). In the timing traces, the black/red bars denote the standard/oddball intervals and the two vertical blue lines indicate a period during which the dynamic array stops at a random time). **B**, Results of Experiment 1a (N = 29, blue curve and squares). **C**, An exemplary stimulus layout of Experiment 3 of set size = 3 (with 6 stationary distractors). Experiment 2 was the same as Experiment 3 except for no stationary distractors on the display. **D**, Results of Experiment 2 (N = 21, squares and blue curve) and 3 (N = 20, triangles and red curve). Error bars are within-subjects SEs [64].

The first paradigm used in Experiment 1a, i.e., a temporal oddball-detection paradigm (TODP), was adapted from the well-established change detection paradigm (CDP) [19] by replacing stationary to-be-memorized object features with dynamic to-be-differentiated temporal intervals. Here, participants were asked to detect an oddball temporal interval which might occur in one of a set of spatial locations (Figure 1A). The stimuli and task of the first paradigm are shown in Figure 1A. In a typical trial, participants first viewed a location array to know how many locations were involved in this trial and where they were. Then a dynamic period, called ‘flashing array’, was followed during which objects (squares) were presented at each of those locations with desynchronized timing. At an unpredictable moment, one object was presented with oddball duration, resulting in an ‘oddball array’. A probe array then came out and participants were required to report whether the oddball occurred in a location circled by an outlined grey ring. For the condition of only one spatial location involved, half trials contain an oddball while the other half not and participants were required to report whether there was an oddball presented in that location.

#### Criteria for trials with excessive eye movements

In Experiment 1a and subsequent experiments involving TODP, eye movement was monitored throughout the experiment by using an eye tracker of Eyelink1000 (SR Research Ltd.) to avoid the usage of saccade-based strategies to scan multi-locations sequentially, e.g. participants switched their gazes rapidly between multiple locations sequentially during the detection of the oddball. The trials of excessive eye movement were operationally defined with following criterion. The average gazes of the whole trial should be confined within a square area (no objects would appear inside this area by the predefined stimulus arrangement), centered at the fixation point with its boarder 3.75 deg from the fixation point. By using this criterion, averagely 6.3% trials were labeled as excessive eye movement trials and thus excluded from subsequent data analysis. The eye tracker was recalibrated after each experimental block of 20 trials.

*In the basic TODP paradigm of Experiment 1a,* the stimuli, 1.2 deg ×1.2 deg black squares (luminance of 5.03 cd/m^2^, measured with a Minolta CS-200 Chromameter photometer), were randomly positioned within a square area, subtending 16 deg ×16 deg, against a white background (luminance of 110.02cd/m^2^). The minimum distance between centers of any two squares was 2 deg and the squares never overlapped with each other. A fixation cross, 0.24 deg width and 0.07 deg thickness, was presented at the center of the display. Each border of the squares was at least 3.75 deg away from the fixation point. Participants were instructed to fixate that cross throughout the experiment.

The standard intervals of the objects were kept constant as 600 ms and the oddball interval 1320 ms, which was determined by an oddball duration experiment (see below). The blank intervals between two temporally neighbored squares were randomly sampled from 800 to 1600 ms. Except for the first square onsets and offsets of all locations, which were synchronized, the timings of all other square onsets and offsets were independently produced. For any trial containing an oddball interval at a spatial location, the oddball square took either the 4^th^, the 5^th^ or the 6^th^ temporal position with equal possibilities in the square sequence of that spatial location. The oddball never appeared at either the 2^nd^ or the 3^rd^ temporal position, aiming to allow the desynchronizations of the timings of the squares across different spatial locations. For the oddball-present trials, a random stop procedure was used in which each trial stopped at a random time between the offset of the oddball and the offset of its immediate next standard interval. For those trials containing no oddballs, e.g. half trials of the single location condition, similar random stop procedure was applied but modified in that the trials stopped between the offset of either the 4^th^, the 5^th^, the 6^th^ standard interval and the offset of its immediate next standard interval. This resulted in an average trial length of 11.8 seconds and matched trial lengths between the oddball-present and oddball-absent trials. Above procedures were adopted carefully in order to avoid any consistent cuing, which might be based on the timing-relationship of multiple objects on the display and be implicitly used by participants during the oddball detection.

Five options of set size (1, 3, 5, 7 or 9 locations) were used. Each set size was repeated 40 times, including 20 ‘Yes’ trials (an oddball appeared in the circled spatial location) and 20 ‘No’ trials (no oddball appeared in the circled spatial location). A gray circle of 2.88 deg radius was used in the response frame to highlight one spatial location. Participants gave their response by pressing one of two response keys. The mappings between ‘Yes’/‘No’ responses and the two response keys were counterbalanced across participants. The whole experiment included 200 trials (10 blocks) in total and different conditions were randomly mixed in the test.

**Figure 2:**
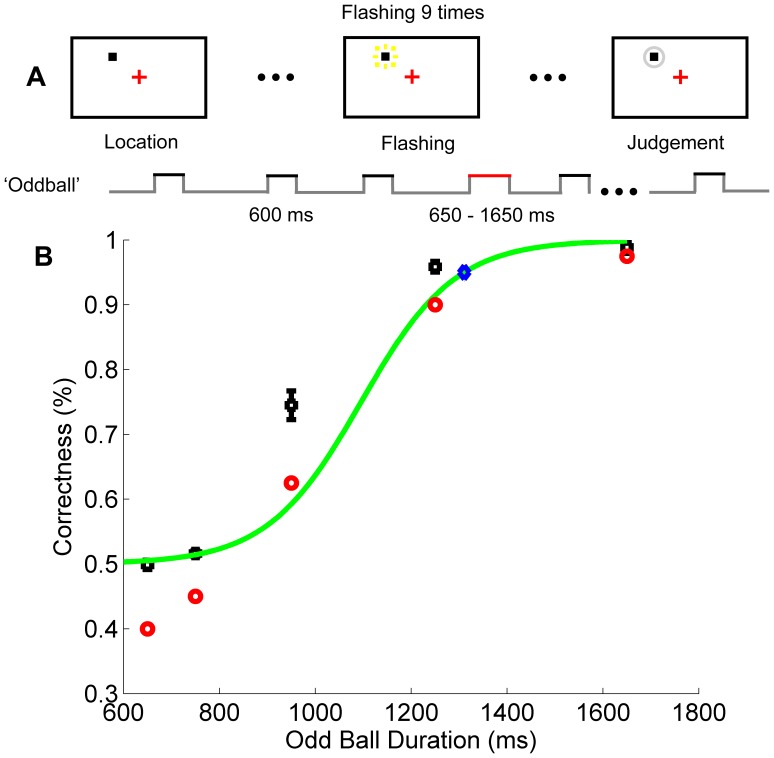
Experimental approach and results of the oddball duration experiment. **A**, Experimental approach of the oddball duration experiment. **B,** Results of the oddball duration experiment. Black squares are mean correctness of 20 participants. Red circles are correctness data from a single participant who had the maximum odd ball duration corresponding to 95% correctness (denoted by a blue diamond) after fitted by a sigmoid model (green curve). Error bars are within-subjects SEs [64].

Before Experiment 1a, an oddball duration experiment was performed to establish the minimum duration that was required for individual participant to detect the oddball interval from a background of the standard intervals (600 ms). This parameter is important because it was chosen to be long enough for participants to differentiate the oddball interval from the standard interval but short enough to avoid unnecessary cuing. Twenty paid participants (16 female, 4 male; mean age = 22.15 years) from CCNU took part in the oddball duration experiment. The stimulus layout of the oddball duration experiment (Figure 2A) was similar to that of Experiment 1a with following exceptions. Firstly, there was only one spatial location in this experiment, i.e., set size always equals to one. Secondly, in half trials, there was no oddball interval. In the other half, an oddball interval occurred, which might take one of five possible values, i.e., 650, 750, 950, 1250 or 1650 ms. Thirdly, the random stop procedure in Experiment 1a was not used here since only one spatial location was involved in this experiment and there was no cuing based on the timing-relationship of multiple objects on the display. In order to avoid that participants identify the oddball via counting, the number of squares at the single location varied from 7 to 11 with equal possibilities and the oddball square, if presented, took a temporal position randomly varied from the 2^nd^ to the 11^th^ in the square sequence. The raw data of the oddball duration experiment of each participant were fitted by a sigmoid curve [20] to calculate the 95% point of correctness (Figure 2B). Considering the fact that participants might have low-level response errors by mistakenly pressing a wrong key, it was more appropriate to take the rule of 95% correctness than the rule of 100% correctness. A similar criterion of 95% correctness was also used in previous literature [21]. The maximum oddball duration corresponding to the 95% correctness of 21 participants was 1320 ms. This critical parameter (1320 ms) was then used in Experiment 1a where multiple spatial locations were introduced to measure the CSTP.

### Results and Discussion

**Figure 3:**
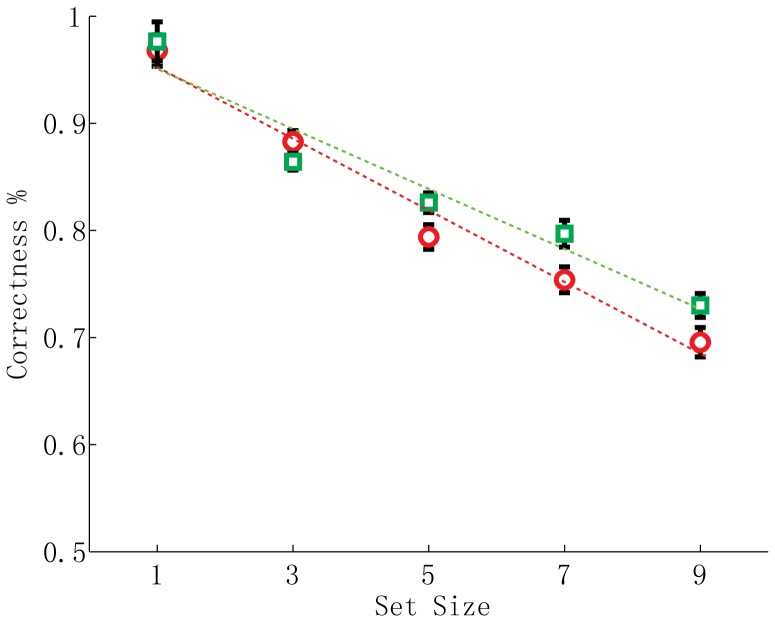
Percentages of correct responses of each set size condition in Experiment 1a (N = 29; red circles) and Experiment 2 (N = 21; green squares) and their linear regression curves. The red curve is for Experiment 1a (slope = −0.034; R^2^ = 0.979; F(3, 1) = 139.227; p<.002) and the green curve for Experiment 2 (slope = −0.028; R^2^ = .941; F(3, 1) = 47.739; p<.007). Error bars are within-subjects SEs [64]. The slope of regression curve in Experiment 1a was not significant different from the slope of regression curve in Experiment 2 (Independent samples t-test; t(48) =  −1.397; p = .169), suggesting the percentages of correct responses in both experiments obeyed a Weber’s law.

With an increase of the set size or the number of involved spatial locations, participants’ performance deteriorated (Figure 3) and capacity estimation reached saturation (Figure 1B). A formula of calculating Cowan’s K [22] was used to estimate the spatial capacity of simultaneous temporal processing in visual modality, similar to the calculation of the capacity of VWM in CDP. Here Cowan’s K was defined as: K = (hit rate+correct rejection rate - 1)×N; N equals to set size. This approach was widely used to measure capacity of the visual working memory [23]–[26], including subsequent working memory experiment in this study.

The results of Experiment 1a (Figure 1B) showed that the Cowan’s K was close to one in the condition of set size 1 (i.e., perfect performance at set size 1), indicating a valid estimation of the required minimum oddball duration. With increase of the set size, the Ks also increase but tend to be saturated.

#### Determine capacity based on Cowan’s K

In the experiments involving TODP and working memory (see below Experiment 1b), capacity was operationally defined as the mean of Cowan’s Ks for a subset of all set size conditions, where K value of each subset member was significantly larger than K values of those conditions that were outside of the subset and had smaller set sizes. Meanwhile the K value of the largest set size condition inside the subset shouldn’t be significantly smaller than those of the rest subset members (This was to correct the calculation of capacity when there is a drop of K value at the largest set size condition). By this definition, the capacity in Experiment 1a was defined as the averaged K values of set size 5, 7, and 9. This was based on the post-hoc pairwise comparisons (after Bonferroni corrections, see Table 1 for the statistical results). Capacity calculations of both multi-temporal processing and working memory (Experiment 1b, see Table 2 for the statistical results) were based on the same principle throughout this study.

**Table 1:** Mean K value differences and p values of pairwise comparisons in Experiment 1a.

Set Size	1		3		5		7	
	MD	p	MD	p	MD	p	MD	p
3	1.354***	.000						
5	1.974***	.000	0.620*	.031				
7	2.602***	.000	1.248***	.000	0.628	.098		
9	2.587***	.000	1.233*	.039	0.613	.532	−0.015	1.00

Note: *p<0.05, ***p<.001.

Mean K value differences in Experiment 1a (Bonferroni corrected pairwise comparisons), indicating that K values of set size 5, 7 and 9 were not significantly different from each other. However, these K values were all significantly larger than those of set size 1 and 3.

**Table 2:** Mean differences of perceived time distortion and p values of pairwise comparisons in Experiment 4.

Number of encoded spatial locations	1L		2Ls		3Ls	
	MD	p	MD	p	MD	p
2Ls	57.2	.199				
3Ls	146.8***	.000	89.7***	.000		
4Ls	151.6***	.000	94.5***	.000	4.8	1.00

Note: *** p<.001.

Mean differences of perceived time distortion in Experiment 4 (Bonferroni corrected pairwise comparisons) indicating that the perceived time distortions of 1L and 2Ls conditions were significantly different from those of 3Ls and 4Ls conditions.

By using above statistical procedure, we operationally defined the capacity of simultaneous temporal processing (CSTP) and found that the average capacity was 3.32 (N = 29, SD = 1.58). These results demonstrated that the capacity of STP is limited for human, in a range between 3 to 4 locations, with a relatively large individual difference (SD = 1.5755).

The result suggested that our visual timing system can co-activate and maintain 3 to 4 independent ‘local clocks’. However, this suggestion seems contradicting with the conclusion of an earlier study by Morgan and colleagues [27] which proposed a single “stopwatch” for duration estimation, similar to a single “ruler” for size estimation (but see [28] for a different opinion on size estimation). The TODP paradigm in our study is also similar to the visual search paradigm used in the Morgan et al.’s study. In the ‘General Discussion’, we gave a thorough comparison and analysis on similarities and differences between these two paradigms and concluded that the claims of these two studies are not necessarily incompatible.

## Experiment 1b

Experiment 1a provides the very first measurement to the capacity of the location-bound simultaneous timing system in vision. According to the result of Experiment 1a, it is likely that our visual timing system can co-activate and maintain 3 to 4 independent ‘local clocks’. This is consistent with recent researches on rats and human participants [4], [8], demonstrating that subjective time estimation in a simultaneous temporal task from millisecond to second level is represented by multiple clocks. Those clocks operate independently to make temporal judgment in a context-dependent way [4]. However, our understanding to how those ‘local clocks’ operate is still scarce. In the next several experiments, we tried to explore the nature of the ‘local clocks’ from two important aspects. First, due to the requirement of our task, the representation of accumulated pulses of each ‘local clock’ need to be maintained and updated online in some memory-like unit. This raises the question of whether the maintenance and updating work in a similar way as the manipulation of representations in the visual working memory. In other words, are those representations of clock-pulses transferred into VWM before the online manipulation? If so, it would be reasonable to predict that the capacity of an individual’s VWM constrains the capacity of the individual’s STP. Alternatively, if the representations of clock pulses are manipulated in units different from those of visual working memory, e.g. some type of pre-working memory registers characterized with easy access, temporary keeping and rapid manipulation, the two capacities should not be constrained by each other.

### Materials and Methods

**Figure 4:**
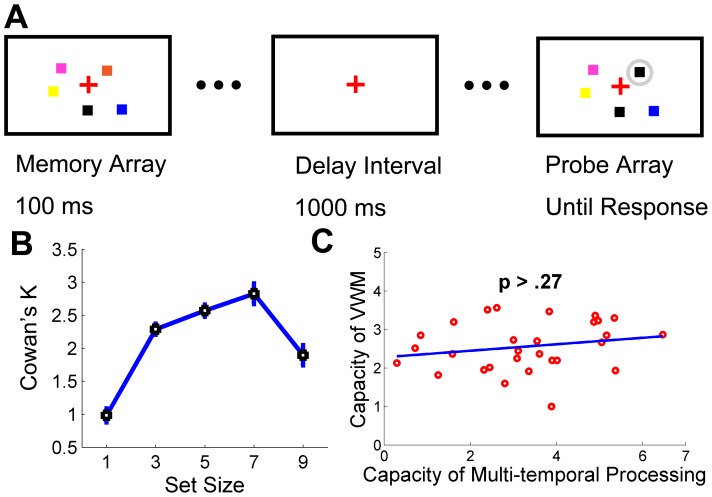
Experimental approach and results of Experiment 1b (the working memory experiment). **A**, Experimental approach of the working memory experiment (an exemplary trial of set size = 5). **B,** The mean Cowan’s Ks of each set size condition in the working memory experiment. **C,** Individual’s working memory capacity was not significantly (p>.27) correlated with his or her spatial capacity of multi-temporal processing. Error bars are within-subjects SEs [64].

In order to answer the above question, the same group of participants was invited into a working memory experiment. The memory array in this experiment (see Figure 4A) consisted of colored square(s) with one out of five set size options (1, 3, 5, 7 or 9). Each square was sampled at random from a set of seven highly discriminable colors (red, blue, violet, green, yellow, black and cyan), and a given color could appear no more than twice within an array. The memory array was presented for 100 ms. Then it was followed by a 1000-ms white blank interval and finally a presentation of the test array which would disappear until the participants made a response. One color of the item in the test array was different from the corresponding item in the memory array on 50% of trials; otherwise the memory and test arrays were identical. Participants were required to judge whether the color square in memory and test array were identical or not. Other aspects of this experiment, such as the size and layout of the squares, were identical as Experiment 1a.

### Results

The mean VWM capacity was 2.56 (N = 29, SD = 0.64) in this experiment (see Figure 4B), a value close to the capacity of 2.8 in Vogel and Machizawa’s 2004 study [23], suggesting a high consistence between these two studies that used similar paradigms. The individual-based correlation between the capacities of VWM and STP was not significant (p = .28) (see Figure 4C), suggesting that these two types of capacities are likely based on different cognitive resources and the representations of accumulated time pluses in each ‘local clock’ are not transferred into the VWM units automatically. This is consistent with a recent research [29], demonstrating a dissociation of visual working memory and the number of encoded spatial locations.

## Experiment 2 and 3

Other factors that might influence the result of Experiment 1a include stimulus crowding and eccentricity. Experiment 1a used a stimulus layout similar to most change detection paradigms in VWM studies, where the eccentricities of different targets and the distances between them were randomized to counterbalance the potential effects of stimulus crowding and eccentricity. We want to ask whether the capacity obtained in Experiment 1a is stable after controlling stimulus properties related with crowding and eccentricity. Experiment 2 & 3 still used the same paradigm, but the spatial layouts of the square(s) were different from Experiment 1a in that all the locations were now positioned along a virtual circle centered at the fixation point (Figure 1C). This stimulus layout allowed the spatial attention to be distributed along a ring-shape area to obtain a constant eccentricity. Meanwhile only nine locations on this invisible ring were used as candidate locations for targets. Thus, the crowding effect was minimized by relatively large and strictly-controlled distances between the targets.

### Materials and Methods

Twenty one paid participants (17 female, 4 male; mean age = 22.14 years) from CCNU took part in Experiment 2. An additional 20 paid participants (17 female, 3 male; mean age = 21.5 years) from CCNU took part in Experiment 3. All aspects of Experiment 2 were the same as Experiment 1a, except that all objects were now positioned along a virtual circle with a radius of 11.3 deg and centered in the fixation (Figure 1C), to control the potential effect of stimulus crowding and eccentricity. The minimum angular distance between any two objects was 40°due to a maximum set size of 9 and all the potential locations were equally distributed along the circle. In a new trial, the nine positions were given a random jitter (maximum rotation angle as 40°) to avoid expectation from the previous trial. Experiment 3 was identical as Experiment 2, except that those locations without objects in Experiment 2 were now occupied by stationary black squares of same size as other squares, i.e., the distracting objects, throughout the trial.

### Results

With above changes applied, the CSTP in Experiment 2 was 3.86 (Figure 1D, N = 21, SD = 1.57, same capacity definition as Experiment 1a), not significantly different from that of Experiment 1a (Independent samples t-test, t(48) =  −1.198, p = 0.237). This showed that the capacity of STP is reliable after controlling crowding and eccentricity-related spatial properties of the stimuli.

A second aspect in our exploration to the nature of the ‘multiple clocks’ was to evaluate what role spatial attention plays in a simultaneous temporal task. In Experiment 3, distracting objects, i.e., the black squares which were always stationary and thus perceptually highly-distinguishable from the dynamic targets, were used (Figure 1C). Independent samples t-tests were used to compare the results of Experiment 2 and 3. We found a significant decrease of Cowan’s Ks in Experiment 3 (Figure 1D) at set sizes of 5 (Difference = −0.87, t(39) =  −2.251, p<.05, SE = 0.39) and 7 (Difference = −1.91, t(48) =  −3.267, p<.003, SE = 0.58), but not at set sizes of 1 (p = .95), 3 (p = .23) and 9 (p = .19) relative to that of Experiment 2. This result demonstrated that the spatial attention was necessary for the ‘local clocks’ to become location-bound. When the allocated attention on the targets was reduced, such as the set size 5 and 7 relative to the set size 3, the binding between clock’s pulse accumulation and its local location was weakened. This can be seen by the mistaken conjunctions between the pulse accumulation of a target and the distracting information from other should-be-ignored locations of the distractors. Those mistaken conjunctions resulted in reduced Cowan’s K at set size 5 and 7 but not at set size 3 where adequate attention was allocated on each of the targets.

## Experiments 4 & 5

Previous research on multiple-object-tracking had demonstrated that humans are able to monitor four or five objects simultaneously [30], [31]. Xu and Chun proposed a two-stage model, including object individuation and object identification, to account for how multiple objects are attended and perceived [32]. This model assumes that capacity limits of various visual phenomenon, such as working memory [33], enumeration [34], and multiple object tracking [35], which are all around four to five objects or regions of interest, are largely due to the constrains of object individuation. The typical explanation to the limitation of object individuation is that our cognitive resource, such as spatial attention, is limited at any given moment and can not cover more than four or five objects simultaneously. Here, we tried to argue that the simultaneous temporal processing is limited in its capacity even without the explicit involvement of the object individuation (at least in its current definition). For that purpose, we designed a second paradigm called spatial splitting of a temporal interval (SSTI) to investigate this issue. The essence of this paradigm was to measure how perceived duration of a physically-fixed temporal interval (1100 ms, standard interval) varied by the number of its encoded spatial locations (1, 2, 3 or 4 location(s)). Here, participants were not required to allocate spatial attention to multi-locations which might trigger the object individuation automatically. There were four critical conditions (denoted as 1L, 2Ls, 3Ls and 4Ls in this paper) in Experiment 4 and three (i.e., 2Ls, 4Ls, and 6Ls) in Experiment 5, corresponding to the number of different spatial locations involved in the standard interval. All the involved locations were stimulated within a time window of several hundred milliseconds.

The idea behind the SSTI paradigm is to test whether only three to four location-bound ‘local clocks’ were activated and maintained during a time window of several hundreds milliseconds. The perceived duration of the standard interval was determined by a summation of ‘clock pulses’ during the sequential presentation of the targets. The activation of any ‘local clock’ will trigger a production of a stream of ‘clock pulses’ at that location, and then contribute to the final duration estimation. However, the opening of a new ‘clock’ at a new location will not close the current ‘clock’ at a different location automatically and immediately. This is due to that the ‘local clocks’ operate independently in vision. The additional activity, due to the delayed closure of the previous ‘local clocks’, would enlarge the summation of the total ‘clock pulses’, as well as the final time estimation. Thus, our prediction is that the time perception of the standard interval in a SSTI paradigm should expand as the number of involved spatial locations increases in a time window of several hundred milliseconds. Also, this effect should saturate at three to four regions of interest due to the limited number of location-bound ‘local clocks’ that can be activated and maintained concurrently in visual modality.

### Materials and Methods

**Figure 5:**
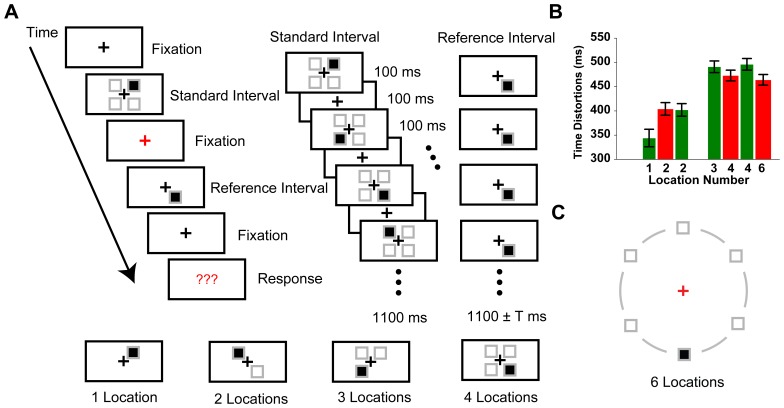
Experimental approach and results of Experiment 4 and 5. **A**, Duration comparison task of the SSTI paradigm. One typical trial of Experiment 4 (upper panel) and its four critical conditions (lower panel). Examples of a standard interval (upper-middle part) and a reference interval (upper-right part) of the 4Ls condition were given. Grey placeholders denoted the involved spatial locations in each critical condition and did not appear in the experimental display. **B**, Results of Experiment 4 (N = 33, green bars) and Experiment 5 (N = 30, red bars). **C**, Stimulus layouts of the six location experiment (a 6Ls condition). Grey circles did not appear in the experimental display. Other aspects were similar to Figure 5A lower panel. Error bars are within-subjects SEs [64].

Thirty three paid participants (21 female, 12 male; mean age = 21.4 years) from CCNU took part in Experiment 4. An additional 30 paid participants (11 female, 19 male; mean age = 21.7 years) from CCNU took part in Experiment 5. In the second paradigm shown in Figure 5A, participants viewed a central fixation followed by two temporal intervals. The standard interval was always 1100 ms, consisted of 1, 2, 3 or 4 to-be-encoded spatial location(s). The reference interval always contained a single location with a variety of durations. Participants were asked to indicate which one of the two intervals was perceived longer in duration.

In the basic SSTI paradigm of Experiment 4, stimulus viewing aperture was a square, subtending 12 deg wide×12 deg high, against a white background. Each trial consisted of a standard interval (1L, 2Ls, 3Ls or 4Ls) and a reference interval.

Each standard interval included 6 sequentially-presented black squares (4 deg wide×4 deg high, each lasted for 100 ms) and 5 100 ms-blank intervals between them. The duration between the onset of the first square and the offset of the last one, which was always 1100 ms physically, indicated the to-be-judged standard interval. The 100 ms blank interval was selected deliberately to minimum the effect of the ‘persistence of vision’ of the immediate-last square, which typically occurs with a blank interval less than 40 ms [36]. Also, the Inhibition of Return (IOR) [37], an effect of impaired detection to objects appearing in previously cued locations relative to locations not previously cued, is unlikely to play a role in this paradigm because a typical IOR needs a SOA of at least 300 ms while the SOA is 200 ms in the present study, and a typical IOR also needs a cue-response paradigm which is absent here. The spatial locations of 4Ls condition were taken from 4 corners of the viewing aperture. The minimum distance between the centers of any 2 squares was 8 deg, a large distance to reduce the effect of apparent motion. Neighboring squares in a standard interval always had different spatial locations. The SOA between any 2 neighboring squares was 200 ms, resulting in a sequential location-stimulation of 5 Hz. In the standard interval, all the involved locations went through once with random order before next going-through. By this arrangement, all the involved locations were presented within the first 100 ms, 300 ms, 500 ms, and 700 ms for the 1L, 2Ls, 3Ls, and 4Ls conditions, respectively. In other words, all the involved locations were stimulated within several hundred milliseconds. In this paradigm, although multiple spatial locations were included, a single object and its occupied single spatial location were involved at any give moment to indicate the continuation of the standard interval. This feature is a critical departure from previous others’ paradigm, for example a study [38] which demonstrated a perceived time expansion induced by simultaneously-presented multiple objects on the display.

The reference interval was composed of one static black square, presenting continuously at one of four locations (Figure 5A, upper-right panel). The spatial position of the reference interval was counterbalanced across different trials. The duration of the reference interval was equally sampled from 7 options, e.g., 200, 800, 1100, 1400, 1700, 2000 or 2600 ms. Here, four of the reference intervals were longer than the standard interval, one was equal to the standard interval, and two were shorter than the standard interval. The reference intervals were 300 ms longer, on average, than the standard interval (1100 ms) because our pilot testing revealed that the 1100 ms standard (dynamic) interval was perceptually overestimated at least 300 ms relative to a 1100 ms (static) reference interval, which is consistent with previous literature [39] demonstrating a time dilation for dynamic stimuli relative to static stimuli. Similar approach of asymmetrical arrangement of the reference stimuli around the standard stimulus was used in recent studies [40], [41]. It is important to note that although this may have affected the distribution of responses, the effect would be constant across all conditions used here. The usage of 4 possible locations within each critical condition (1L, 2Ls, 3Ls or 4Ls) of both standard and reference intervals was counterbalanced across trials to make sure that each location had equal chance to be used. Thus, the effect of apparent motion was equalized across the conditions of 2Ls, 3Ls and 4Ls. Participants were instructed to keep gazing at a fixation cross throughout the experiment.

The standard and reference stimuli were sequentially presented within the two intervals, with a random ISI (Inter-Stimulus Interval) varying between 500 ms and 1000 ms. The order of the standard and the reference intervals in a trial was counterbalanced across different trials. The participants’ task was to indicate which interval (the first or the second) contained duration that seemed to last longer by pressing one of two response keys. Each level of duration of the reference interval was repeated for 24 times, resulting in a total of 672 trials (168 trials for each condition of 1L, 2Ls, 3Ls or 4Ls). Different conditions were randomly mixed during testing.

All aspects of Experiment 5 were identical as Experiment 4, except that 1L and 3Ls conditions were replaced by a new condition, i.e., 6Ls, where six potential locations were used and all of them positioned along a virtual circle centered in the fixation with a radius of 5.66 deg (Figure 5C). The minimum angular distance between any two locations was 60°due to that all the six locations were equally distributed along the circle. In any new trial, the six positions were given a random jitter (with a rotation angle between 0°to 60°) to avoid expectation from the previous trial. There were 3 critical conditions in Experiment 5, i.e., 2Ls, 4Ls and 6Ls.

### Results

In Experiment 4, and 5 that involved the measurement of perceived time distortions, a sigmoid curve was fitted on each individual’s data to calculate the PSE (Point of Subject Equality) at each critical condition (1L, 2Ls, 3Ls, 4Ls, or 6Ls). Participants had a 50% percentage to report a ‘longer’ standard interval at the PSE, indicating that the standard and the reference intervals were perceptually equal in duration. The perceived time distortions were subsequently defined as the differences between the PSEs and the standard intervals (1100 ms).

As predicted, Experiment 4 demonstrated an effect of number of encoded spatial locations on time estimation (F (3, 96) = 21.932, p<0.001). With the number of the encoded spatial locations increased, the standard interval was perceived longer in duration (Figure 5B). Particularly, the 1L and 2Ls conditions were qualitatively different from the 3Ls and 4Ls conditions in that the mean perceived time distortion of 1L and 2Ls were significantly smaller (Difference =  −121 ms, t(32) =  −7.424, p<.001) than that of 3Ls and 4Ls conditions. The hypothesis that the location-number-related effect reaches a plateau at around three to four spatial locations received further support from Experiment 5, where no significant difference was found between 4Ls and a condition with more location number, i.e., 6Ls (Figure 5C), though time distortions of both conditions were larger than that of 2Ls condition (p<.008 for 4Ls and p<.03 for 6Ls, Bonferroni corrected). This result supported the idea that only 3 to 4 location-bound ‘local clocks’ are activated and maintained during a time window of several hundreds milliseconds. Our finding implicates that the hypothetic ‘object individuation’ [32] might need a modification, e.g., to include the ‘multiple clocks’ maintained during a time window of several hundreds milliseconds, in order to explain the capacity of simultaneous temporal processing.

## General Discussion

Our results, based on two novel and complementary paradigms, indicate consistently that the capacity limit of simultaneous temporal processing in vision is around 3 to 4 spatial locations and the capacity of STP is not constrained by the capacity of visual working memory (VWM). Moreover, the binding of the 3 to 4 ‘local clocks’ and their specific location is undermined by bottom-up competition of spatial attention, indicating that the time-space binding is resource-consuming. The second paradigm confirms further that the limited number of location-bound ‘local clocks’ are activated and maintained during a time window of several hundreds milliseconds.

### Distributed Attention vs. Focal Attention

In an earlier study, Morgan and colleagues (2008) [27] explored whether the different thresholds of temporal oddball-discrimination was related with set size in a visual search paradigm. They asked their participants to report whether a single “odd duration” was shorter or longer than the other distractor durations inside a visual search array with a set size of 2, 4, 6 or 8. The temporal orders of the onsets of the odd duration and the distractors were randomized, similar to the onset timing of the oddball and standard squares in our TODP paradigm, though their stimuli were all horizontal lines staggered in space. The usages of the temporal oddball and multiple distractors were very similar in these two paradigms. However, Morgan and colleagues found that the precisions, i.e. Weber fractions, of discriminating an odd duration from a set of distractors were affected by the set size and concluded that a centralized supramodal clock, i.e. a single “stopwatch”, was used for duration estimation. Although our results suggested 3–4 independent ‘clocks’, our conclusion is not necessarily contradicted with their conclusion because our TODP paradigm differs from Morgan and colleagues’ visual search paradigm (2008) [27] in significant ways.

First, by using an eye tracker our TODP paradigm controlled eye movements and elicited a broadly *distributed attention* over the entire stimulus viewing aperture, whereas the visual search paradigm of Morgan and colleagues allowed multiple fixations and shifts of *focal attention* between different spatial locations during the search to the odd duration (0.5 s, 2 s and 8 s). Here, focal attention means to concentrate on small areas for visual processing [42]. It has been well documented in the visual search literature [28], [42]–[45] that visual search performance was qualitatively different between *distributed* attention and *focal* attention. For example, it was revealed [43] that a broadly distributed attentional allocation is sufficient for participants to search an odd-coloured target in a parallel way with unlimited capacity, i.e. a parallel process is operating over large areas. In contrast, when participants were asked to use saccades to indicate the odd-coloured target, a serial search by shifting focal attention to each target was performed. This implied that the goal-directed saccades are concurrent with the shifts of focal attention even in simplest visual search tasks. Thus, it is not surprising that a serial-like process was found in Morgan and colleagues’ paradigm, while a parallel-like process was elicited in our TODP paradigm. Second, task difficulty or more specifically the feature distance between a target and its background might also contribute to the differences between these two paradigms. By using the oddball duration experiment we selected a large feature distance between the oddball (1320 ms) and the standard intervals (600 ms), i.e. corresponding to correctness in-between 95% to 100%. Morgan and colleagues’ study measured a discrimination threshold of 82.5% correct response. To reduce performance to threshold, their paradigm used very similar targets and distractors, whereas the targets and distractors in our paradigm were more different. Based on the suggestion of previous literature [46], a task of a larger distance in feature space, such as the task of our TODP paradigm, is more likely to elicit a parallel process than the task of Morgan and colleagues.

Another factor that might be relevant here is the difference between a *detection* task and a *discrimination* task. Sagi & Julesz (1985) [21] proposed that to detect a feature of a target that is *different* from its background involves different attentional process compared with to discriminate the *direction* of the feature gradient of a target from its background. The former involved a parallel (preattentive) process, while the latter involved a serial attentive process. In its definition, our TODP paradigm was a typical detection task (to detect the occurrence of a ‘longer’ duration), while Morgan and colleagues’ paradigm was more likely to be a discrimination task involving feature directions (to discriminate a ‘shorter’ or ‘longer’ oddball) in each trial.

Taken together, we proposed that the differences in task demands, task difficulties, and performance measurements, might interact in a complex way and elicit essentially different attentional processes for these two paradigms, i.e. distributed attention in our TODP paradigm and focal attention in Morgan and colleagues’ paradigm. The former might induce parallel-like process by *distributed* attention, while the latter might elicit serial-like process by *focal* attention. In that sense, our conclusion of the 3 to 4 concurrent ‘local clocks’ in vision are not necessarily incompatible with Morgan and colleagues’ claim that a single “stopwatch” is operating for duration estimation. In fact, it is very likely that both claims revealed different aspects of how same amount of a limited-capacity is dynamically allocated under different situations, i.e. *distributed* attention vs. *focal* attention.

### Constraints from Spatial Processing

In visual modality, spatial encoding and temporal processing are closely linked. The classic Kappa effect [47] and Tau effect [48] indicated dynamic interactions between spatial judgment and time perception. A major theoretic development in the past decade is Walsh’s theory [49] which sheds new light on the relationship between spatial encoding and temporal processing. According to this theory called ATOM (A Theory of Magnitude), processing of time, space, or quantity all shares a common magnitude system and overlapped neutral substrate, e.g. the inferior parietal cortex. The present study adds new evidence to a growing body of literatures that the temporal processing in visual modality is constrained by spatial processing.

The precise estimation to the duration of a visually-presented object, such as the square in the present study, relies on continuous online updating and monitoring to the object’s spatial feature, such as the brightness. Thus, it is justifiable that the spatial processing, particularly the quality and frequency of the ‘spatial feature monitoring’ can constrain the object’s temporal representation, i.e., the accumulated time ‘pulses’. The construction of the temporal representation relies on a successful conversion of encoded information from spatial domain into temporal domain. The exact nature of this information conversion is not yet clear, partially due to the debate on how temporal pulses are accumulated, i.e., by a linear or nonlinear way [50], [51]. Adaptation studies [9]–[11] implied the existence of location-based timing system in vision. However, it is not yet known how spatial processing imposes constraints on this temporal processing. Our findings of the present study demonstrate clearly that this location-based timing system is subject to a spatial-processing-related bottleneck, i.e., at any given moment or during any interval of hundred milliseconds the information conversion from spatial domain into temporal domain can only be maintained concurrently at 3 to 4 spatial locations.

It is worth to note that this bottleneck is unlikely solely determined by low-level factors, such as crowding and eccentricity-related effects, since our Experiment 2 demonstrated a relatively stable capacity of STP after controlling those low-level spatial properties. Instead, this bottleneck is most likely located at relatively higher level of visual hierarchy, such as those linked with limited resource of spatial attention. This is confirmed by our Experiment 3, which indicates that certain amount of the allocated spatial attention is necessary for the successful conversion of information from spatial domain into temporal domain. Considering the change of the general pattern of distributed attention from Experiment 2 to 3, when the averaged amount of allocated attention at each of the relevant spatial locations was reduced by the introduction of a group of should-be-ignored static distractors, i.e. with the presence of bottom-up attentional competition, the temporal estimations of those relevant locations were greatly undermined. It is an open question for future study to explore whether the attentional competition also invokes interference to spatial processing. Similar effect was observed in the situation of multiple-object-tracking [52] in that the changes of the moving objects’ spatial features, such as color or shape, is surprisingly blinded and miss-reported when attentional resource is directed to the tracking of the objects’ identities.

### Multiple Capacities in Vision?

An interesting observation in the present study is that the capacity of STP is not constrained by an individual’s capacity of VWM. This implies that multiple capacities, rather than a universal single one, exist in visual modality. Indeed, similar dissociations of separate capacities in vision had been reported before. For example, Hyde & Wood suggested [53] that in nonverbal numerical processing the approximate number system (ANS)-based numerical representations are not constrained by the capacity of VWM since those representations either only take one VWM slot or do not enter into VWM automatically. Other literature [54] demonstrated that the attention-related capacity, including multiple object tracking (MOT), detection of RSVP and nonverbal enumeration etc, is qualitatively different from a VWM-related capacity, such as that measured in a change detection task of VWM. The attention-related capacity can not be reducible to a VWM-capacity. Instead, VWM-capacity relies on multiple factors, including visuospatial attention; a central, amodal supervisory process; and local stage-specific operations. One critical similarity between STP of our task and nonverbal numerical processing is that both of them are subject to a spatial attention-based bottleneck. This leads to why the capacity of STP in the present study is not constrained by an individual’s capacity of VWM.

As suggested by our second paradigm, the STP capacity is unlikely dependent on the object individuation, i.e., the activation of a parallel individuation system [55], [56]. This is further confirmed by the fact that we found reliable set size-correctness relationships in Experiment 1a and 2. In those two experiments, the relationship between the correct response and the set size all obeyed Weber’s laws (see Figure 3). That is to say, the size of the variance introduced into the temporal estimation in our STP task is positively correlated with the number of spatial locations that need to be attended to. This indicates that at all levels of different spatial locations our participants did not simply select 3 to 4 objects first and mask the rest of all the other objects. Instead, the cognitive resource is distributed across all the possible spatial locations in a parallel way with limited capacity. Particularly, when spatial attention is further spread over the visual scene by introducing more to-be-processed spatial locations, more variance is added into the numerical estimation at each location and thus increases the response error. The obedience to Weber’s law is a hallmark of the approximate number system in nonverbal numerical processing rather than that of the subitizing which is likely based on a parallel individuation system [55], [56]. Our result adds new evidence that nonverbal numerical processing and temporal estimation share common cognitive principles and even neutral basis. This is supported by a recent neuroimaging study [57], indicating both numerosity and duration estimations share a common right fronto-parietal network. All above facts can be justified in the context of an accumulator model [13] in that both numerosity and time are likely based on a common accumulator that is working under two different modes.

A recent study on nonverbal numerical processing [53] demonstrated that it is the allocation of spatial attention rather than the absolute magnitude of quantity itself that determines which system, a parallel individuation system or a ANS-like ‘numerical magnitude’ system, should be deployed in a given visual scene. When objects are too numerous to be selected simultaneously or spatial attention is distributed across the visual field, the visual system will invoke the ANS system to perform approximate estimation to numerical information, even to small numbers (<3), in the visual scene. A STP task in vision, such as the TODP paradigm here, requires constant attentional consumption and distributed attention. Thus, it is not surprising that visual system automatically invoke an ANS-like system to estimate the number of accumulated temporal pulses at each attended location. This explains why we did not get a Cowan’s K close to 3, i.e., close to 100% correct performance (as predicted by a parallel individuation-like system), at the condition of set size 3 in Experiment 2 when this set size is well below the capacity of STP (3.86). Further data collections are needed in order to explore whether the counterpart of ANS in the domain of time perception, i.e., a plausible ATS (Approximate Time System)-like system, exists in simultaneous temporal processing.

### Neural Basis of STP

Previous neurophysiologic studies provided clues on the possible neural basis of the capacity of simultaneous temporal processing. In one hand, a study [58] demonstrated that two distinct neuron populations in the striatum (STR) of rats were activated differentially by rewards of two criterion durations (10 s vs. 40 s), implicating two ‘clocks’ in the striatum. On the other hand, other study showed that the prefrontal cortex (PFC) was necessary in terms of dynamically allocating attention to multiple timed stimuli [59]. These results are compatible with our findings that ‘multiple clocks’ can coexist and the bindings between the ‘multiple clocks’ and specific locations are attention-dependent. A growing body of researches on human and monkey participants had demonstrated that a distributed network [60]–[63], including basal gangalia, cerebellum, PFC, pre-supplementary motor area (preSMA) and supplementary motor area (SMA), involves in multiple aspects of temporal processing depending on the task requirements, i.e., motor vs. perceptual and sub-second vs. supra-second judgments. Further neural imaging studies on human by using sub-second intervals are necessary to investigate where a potential bottleneck, corresponding to the capacity of simultaneous temporal processing, lies in the cortico-striatal circuits and its exact role in our ability to time multiple events simultaneously.

In conclusion, we found that the temporal processing in visual modality is constrained by spatial processing when time estimations are based on information of encoded spatial locations. Specifically, the present study provide converging evidence to support that our capacity of simultaneous temporal processing is limited at around 3 to 4 spatial locations in visual modality. This spatial capacity in vision is subject to the allocation of spatial attention but not constrained by the capacity of working memory and is unlikely be determined by the object individuation. This conclusion receives highly consistent supports from two novel and complementary paradigms.

A demonstration of stimuli used in this study can be found at http://visionlab.byethost7.com/.
